# Associations Between Sexual Behavior Stigma and HIV Risk Behaviors, Testing, Treatment, and Infection Among Men Who have Sex with Men in Ukraine

**DOI:** 10.1007/s10461-023-04182-1

**Published:** 2023-10-04

**Authors:** Ben Alvey, Jack Stone, Tetyana Salyuk, Ezra J. Barzilay, Ivan Doan, Peter Vickerman, Adam Trickey

**Affiliations:** 1 Population Health Sciences, University of Bristol, Bristol, UK; 2 Health Protection Research Unit in Behavioural Science and Evaluation at University of Bristol, Bristol, UK; 3 Alliance for Public Health, Kyiv, Ukraine; 4 Centers for Disease Control, Kyiv, Ukraine

**Keywords:** IBBS, Homosexual, Bisexual, Antiretroviral therapy, Stigma, Ukraine

## Abstract

Stigma toward same-sex behaviors may be a structural driver of HIV epidemics among men who have sex with men (MSM) in Eastern Europe and has been linked to adverse HIV-outcomes elsewhere. We explored associations between sexual behavior stigma with HIV risk behaviors, testing, treatment, and infection. From November 2017 to February 2018, MSM across 27 Ukrainian cities were recruited to cross-sectional surveys using respondent driven sampling. Eligible participants were cisgender males aged ≥ 14 years residing in participating cities that reported ≥ 1 sexual contact with another man in the prior 6 months. Participants self-reported experience of stigma (ever) and various HIV-outcomes and were tested for HIV antibodies. Regression models were used to explore associations between three sexual behavior stigma variables with demographic and HIV-related variables. Of 5812 recruited cisgender MSM, 5544 (95.4%) were included. 1663 (30.0%) MSM reported having experienced stigma due to being MSM from family and friends, 698 (12.6%) reported anticipated healthcare stigma, and 1805 (32.6%) reported general public/social stigma due to being MSM (enacted). All forms of stigma were associated with heightened HIV risk behaviors; those experiencing stigma (vs not) had more anal sex partners in the prior month and were less likely to have used condoms during their last anal intercourse. Stigma was not associated with HIV infection, testing, or treatment variables. A sizeable proportion of Ukrainian MSM reported ever experiencing stigma due to being MSM. MSM that had experienced stigma had higher odds of HIV sexual risk behaviors. Further study using longitudinal designs is required to determine causality.

**Supplementary Information:**

The online version contains supplementary material available at 10.1007/s10461-023-04182-1.

## Introduction

Eastern Europe and Central Asia (EECA) currently reports the fastest growing HIV epidemic of any region worldwide [1]. Within the region, Ukraine has the second largest HIV burden with an estimated 250,000 people living with HIV (PLHIV) [2]. HIV prevalence is high among the estimated 180,000 MSM living in Ukraine, estimated to be 7.5% in 2019 [2]. In Ukraine, same sex sexual activity was legalised in 1991 after the dissolution of the Soviet Union. Although Ukraine is one of the few EECA countries to have anti-discrimination laws concerning sexual orientation, an ‘anti-propaganda laws’ has been proposed that would impose administrative and criminal liability to ‘propaganda of homosexuality’ [3].

Individual-level HIV risks [e.g. high transmission probability during unprotected anal intercourse (UAI)] and network-level risks (e.g. high prevalence within sexual-networks) may be compounded by community-level and structural-level risks such as stigma to increase HIV-risk among MSM [4]. Stigma has been conceptualised as a social process labelling individual or groups as less valuable than others within a larger community based on actual or perceived characteristics, resulting in adverse-experiences, reduced opportunity, and reduced wellbeing [5]. Different forms of stigma have been characterised [6]. Perceived stigma is a person’s belief that others treat or think about individuals with a stigmatised characteristic differently. Enacted stigma is the explicit experience of mistreatment because of a stigmatised characteristic. Anticipated stigma is the expectation of future stigma experiences. Internalised stigma is a person’s acceptance and application of negative feelings to oneself because of one’s stigmatised characteristic [6].

Multiple stigma constructs are relevant to MSM, including sexual behavior stigma, defined as stigma based on one’s sexual practices [6], and stigma based on sexual-orientation, gender identity and HIV status (HIV stigma) [7]. These stigma constructs may also co-occur and interact within individuals or groups of MSM, termed intersecting stigma [6].

Intersecting sexual behavior, sexual orientation, and gender identity stigmas have been linked with adverse HIV-related outcomes among MSM which impede HIV prevention, diagnosis, and treatment [6, 8]. At the individual-level, these intersecting stigma have been associated with UAI, fear and avoidance of seeking health care, and reduced HIV testing in the US, Namibia, and South Africa [9–12]. Within Europe, MSM living in countries with higher levels of stigma are less likely to use testing services, discuss their sexuality in testing services, and have diagnosed HIV infection, but are more likely to have greater unmet prevention needs, a lack of HIV transmission knowledge, and higher levels of sexual risk behavior [13]. High levels of sexual risk behavior could lead to increased risk of HIV infection among MSM [14, 15].

Initiatives (termed societal enablers) to remove punitive laws and policies and to reduce stigma and discrimination are a central focus of the 2021 UN declaration to eliminate AIDS by 2030, including a target to invest $3.1 billion in societal enablers [16]. However, evidence on the prevalence and effects of stigma among MSM—and indeed other key populations—is lacking in many countries and regions and unable to adequately inform an evidence-based response. Much of the current data on stigma among MSM is on stigma due to having HIV [17, 18], while stigma relating to sexual behaviors of MSM irrespective of HIV status is comparatively neglected [6]. Moreover, it is critical to study sexual behavior stigma in countries with sustained HIV epidemics alongside continuing high stigmatisation of MSM. Multi-country studies considering relationships between HIV and stigma towards MSM include data from Eastern Europe [13, 19]. However, these either consider structural-level sexual behavior stigma [13], or measured stigma relating to sexual orientation [19]. Measures of stigma based on sexual orientation, such as homosexuality, may inadequately apply to MSM not identifying as homosexual [8]. No studies known to the authors explored associations between community-level sexual behavior stigma and HIV-related outcomes in Eastern Europe.

In this context, this study aims to explore whether there are associations between sexual-behavior stigma with concealment of sexual orientation, high-risk sexual behaviors, HIV testing behaviors, HIV prevalence, and HIV treatment, among MSM in Ukraine, using data from a national survey of MSM from 2017 to 2018.

## Methods

### Integrated Bio-behavioral Survey (IBBS) Data

We used data from a nationwide cross-sectional IBBS of MSM in Ukraine undertaken by the Alliance for Public Health, a non-governmental organisation (NGO) working to control HIV in Ukraine [20]. Data collection occurred between November 19th 2017 and February 3rd 2018. Eligible participants were MSM that reported at least one sexual contact with another man in the past 6 months. These MSM must have resided in 27 participating cities (Supplementary Table 1), were ≥ 14 years old, and consented to completing a questionnaire, providing a dried blood spot sample, and participating in HIV testing. The surveys were carried out in different venue types, mostly rented office blocks, AIDS centres and, in some cases, the offices of non-governmental organisations. HIV testing was performed in each survey to determine a respondent’s HIV status using Profitest HIV test [NEW VISION DIAGNOSTICS Inc. (Bayamon, Puerto Rico)] and confirmed using SD Bioline HIV 1/2 3.0 [ABBOTT LABORATORIES (Chicago, IL)]. Rapid testing of the blood samples was carried by experts of regional AIDS centers or other authorized medical institutions, with dry blood samples collected for further analysis by the National Reference Laboratory. Participants were recruited using respondent driven sampling (RDS) [6, 20]. We excluded transgender individuals from this analysis, as an analysis of stigma among transgender individuals has been planned separately.

### Measures

The IBBS included survey items asking participants whether they had ever experienced any of 13 stigma-related situations relating to someone knowing or finding out that they have sex with men. Three sexual behavior stigma measures were created from these individual items based on methods developed by Augustinavicius et al. [8] to separate the types of stigma and who stigmatised them. These measures of stigma have not been validated for Ukrainian MSM, although they have been used previously in the United States and sub-Saharan Africa [8, 15]. These binary measures were:

- “Stigma from family and friends”
- “Anticipated healthcare stigma”
- “General enacted public/social stigma”

The individual questions and how they are grouped into the binary variables above are listed in Supplementary Tables 2 and 3. A person was considered to have experienced one of these grouped measures of stigma if they experienced any of the individual items within the grouped measure of stigma. For “General social stigma” we chose to remove the item “Afraid to be in public because they were MSM” from this category, so that the category solely contained enacted stigma, rather than anticipated stigma. The reliability of these composite measures of stigma was assessed using Cronbach’s Alpha.

### Data Analysis

Stata 16.1 was used for all analyses. Observations with missing data on stigma items were excluded. RDS weights were avoided because of insufficient consensus regarding their validity in regression models and were therefore not included in any analyses for consistency [21]. Variables of interest were cross tabulated against each stigma measure. Denominators may vary between cross-tabulations if data was missing on a given variable of interest.

### Socio-demographic Characteristic Predictors of Stigma

We examined associations between socio-demographic characteristics, as independent variables, with each of the stigma measures as dependent variables using mixed-effects logistic regression models with city as the random-effect. In unadjusted and adjusted analyses, we tested whether age (per 10 year increase), education level (categorical), ever having been imprisoned (versus not), marital status (categorical), cohabitation status (categorical), sexual orientation (categorical), sexual-orientation concealment (categorical), and reporting being an NGO client [22, 23] (versus not) were associated with the three stigma measures. Being an NGO client was defined as reporting having an NGO membership card. Monthly income was excluded due to high missingness for this variable.

### Stigma Predicting Demographics, Sexual Behaviors, and HIV Testing, Status, and Treatment

We tested associations between having ever experienced each stigma measure, as the independent variable, with various HIV risk-behavior, testing, and HIV treatment variables as dependent variables (Table 3). These variables were: concealing their sexual orientation from everyone, being a client of NGO which provides prevention services to MSM (as somebody that receives stigma may look to an NGO for support, or being a client of an NGO may mean that person is more likely to be ‘outed’), age at first oral or anal sex with a man (years), having had anal sex with a man in the last 6 months, number of anal intercourses in last 30 days, number of male anal sex partners in last 30 days, reporting always using a condom for male anal sex during last 30 days, reporting using a condom for last anal sex with a man, having ever been renumerated for sex, reporting having group sex in the last 6 months, reporting having chemsex in last 30 days [24], considers knowing sexual partners’ HIV status to be very important, considers disclosing HIV status to sexual partners be very important, being aware of last permanent partner’s HIV status, ever being tested for HIV, being tested for HIV in the last year, self-reporting having HIV, testing HIV positive, reporting being registered at AIDS centre, and reporting receiving ART.

For binary dependent variables we used mixed-effects logistic regression models with city as the random-effect. For numeric dependent variables we used mixed-effects linear regression models if the variable was normally distributed (age of first sexual contact with a man) or mixed-effects negative binomial regression models if the variable had a negative binomial distribution (number of male anal intercourses in last 30 days and number of male anal sex partners in last 30 days). Unadjusted and adjusted models, adjusting for age, education level, imprisonment history, marital status, cohabitation status, sexual orientation, sexual orientation concealment, and reporting being an NGO client, were assessed.

We hypothesised sexual orientation concealment and NGO client status could either result from or induce stigma. Therefore, these variables were tested firstly as independent variables with stigma as the dependent variable, and secondly as dependent variables with stigma as the independent variable. In adjusted analyses sexual orientation concealment and NGO client status were not adjusted on themselves.

When testing associations of stigma with being registered at an AIDS centre and with receiving ART the denominator was participants testing HIV seropositive, giving a sample size of only 293. To enable model convergence, the models of registration at an AIDS centre and receiving ART were adjusted on fewer explanatory variables. These variables were age (which associates with both sexual behavior stigma and HIV outcomes in other settings [25]), plus additional variables chosen using a stringent cut-off of p < 0.01 in univariable analyses: imprisonment, marital status, cohabitation status, concealment, and NGO membership.

## Results

Of the surveyed cisgender MSM, 5544 of 5812 (95.4%) had complete data on stigma variables and were included in our analyses. Table 1 and Supplementary Table 4 show sample characteristics of included MSM stratified by whether they ever experienced each stigma measure. 1663 (30.0%) MSM reported having experienced enacted stigma from family and friends, whilst 698 (12.6%) reported anticipated healthcare stigma, and 1805 (32.6%) reported general, enacted social stigma. Overall, 2577 (46%) of MSM reported experience of at least one of these stigma constructs. Each of these composite measures of stigma was deemed to be reliable based on the Cronbach’s Alpha scores of over 0.6: 0.6106 for stigma from family and friends, 0.7684 for anticipated healthcare stigma, and 0.6407 for general, enacted social stigma. Overall, the median age of included MSM was 27 with an interquartile range (IQR) of 21–35. Testing seropositive for HIV were 286 MSM (5.2%), but just 141 (49.3%) of these self-reported as being aware they had HIV.

**Table 1:** Characteristics of MSM by whether they have experienced each stigma measure (N and column percentages)

Variable	“Stigma from family and friends”				“Anticipated healthcare stigma”				“Enacted general social stigma”			
	Never		Ever		Never		Ever		Never		Ever	
*n* = *5544 unless otherwise specified*	(n = 3881, 70.0%)		(n = 1663, 30.0%)		(n = 4846, 87.4%)		(n = 698, 12.6%)		(n = 3739, 67.4%)		(n = 1805, 32.6%)	
Education												
Secondary	1670	43.0%	709	42.6%	2047	42.2%	332	47.6%	1544	41.3%	835	46.3%
Incomplete higher	946	24.4%	408	24.5%	1188	24.5%	166	23.8%	954	25.5%	400	22.2%
Complete higher	1265	32.6%	546	32.8%	1611	33.2%	200	28.7%	1241	33.2%	570	31.6%
Official marital status												
Never married	3122	80.4%	1383	83.2%	3945	81.4%	560	80.2%	2996	80.1%	1509	83.6%
Officially married	252	6.5%	41	2.5%	259	5.3%	34	4.9%	231	6.2%	62	3.4%
Divorced or widowed	507	13.1%	239	14.4%	642	13.3%	104	14.9%	512	13.7%	234	13.0%
Cohabitation status												
Live with parents/relatives	1524	39.3%	608	36.6%	1870	38.6%	262	37.5%	1467	39.2%	665	36.8%
Live alone	1552	40.0%	668	40.2%	1933	39.9%	287	41.1%	1474	39.4%	746	41.3%
Live with male partner	557	14.4%	349	21.0%	791	16.3%	115	16.5%	564	15.1%	342	19.0%
Live with female partner	248	6.4%	38	2.3%	252	5.2%	34	4.9%	234	6.3%	52	2.9%
Ever imprisoned												
Don’t report ever being imprisoned	3789	97.6%	1588	95.5%	4719	97.4%	658	94.3%	3651	97.7%	1726	95.6%
Previously imprisoned	92	2.4%	75	4.5%	127	2.6%	40	5.7%	88	2.4%	79	4.4%
Sexual orientation												
Homosexual	2443	63.0%	1140	68.6%	3182	65.7%	401	57.5%	2326	62.2%	1257	69.6%
Bisexual or other	1438	37.1%	523	31.5%	1664	34.3%	297	42.6%	1413	37.8%	548	30.4%
Sexual orientation concealment^a^												
Did not report any concealment	262	6.8%	212	12.8%	426	8.8%	48	6.9%	257	6.9%	217	12.0%
Partial concealment	2387	61.5%	1095	65.8%	3079	63.5%	403	57.7%	2271	60.7%	1211	67.1%
Conceal from everyone	1232	31.7%	356	21.4%	1341	27.7%	247	35.4%	1211	32.4%	377	20.9%
Client of MSM-focused NGO												
Does not report as being an NGO client	2871	74.0%	1145	68.9%	3493	72.1%	523	74.9%	2798	74.8%	1218	67.5%
NGO client	1010	26.0%	518	31.2%	1353	27.9%	175	25.1%	941	25.2%	587	32.5%
Self-reported HIV status (*of n* = *3625 ever HIV-tested*)												
HIV negative self-report	2080	83.6%	911	80.1%	2662	83.8%	329	73.6%	1998	83.8%	993	80.0%
HIV positive self-report	101	4.1%	40	3.5%	117	3.7%	24	5.4%	85	3.6%	56	4.5%
Declined to disclose HIV status	306	12.3%	187	16.4%	399	12.6%	94	21.0%	301	12.6%	192	15.5%
HIV antibody test result												
Negative	3675	94.7%	1583	95.2%	4604	95.0%	654	93.7%	3562	95.3%	1696	94.0%
Positive	206	5.3%	80	4.8%	242	5.0%	44	6.3%	177	4.7%	109	6.0%
Median age (interquartile range)	27 (21–35)		27 (21–35)		27 (21–35)		27 (21–36)		27 (21–35)		27 (21–34)	

*MSM* men who have sex with men; *NGO* non-governmental organisation; *HIV* human immunodeficiency virus

^a^The question available in the questionnaire was “Do you suppress the fact that you have sex with men?”. The possible answers were “Suppress this from everybody”, “Do not suppress this and is ready to say it anywhere”, and “Do note suppress this, but I will not talk about this first”

### Socio-demographic Characteristic Predictors of Stigma

Table 2 reports unadjusted and adjusted odds ratios of each stigma measure for socio-demographic characteristics, where the measures of stigma are the dependent variables. In adjusted analyses, MSM with higher levels of education had reduced odds of anticipated healthcare stigma and general social stigma. MSM that were divorced or widowed had reduced odds of anticipated healthcare stigma compared with those that had never been married. Compared with MSM that live alone, those that live with a male partner had higher odds of stigma from family and friends, whilst those living with a female partner had lower odds. MSM living with parents/relatives or a female partner had lower odds of general social stigma than those living alone. MSM that had ever been imprisoned were more likely to report ever having experienced all three stigma measures. Compared to MSM reporting homosexual orientation, those reporting bisexual or other sexual orientation had higher odds of anticipated healthcare stigma but lower odds of general social stigma. For sexual-orientation concealment, MSM with higher levels of concealment had reduced odds of stigma from family and friends and general social stigma, and increased odds of anticipated healthcare stigma. MSM that were clients of an MSM-focused NGO organisation had increased odds of reporting general social stigma than non-members.

**Table 2:** Unadjusted and adjusted odds ratios of reporting ever (versus never) experiencing each stigma measure for socio-demographic characteristics

Independent variable	Stigma measures (dependent variable)					
	“Stigma from family and friends” (ever vs never)		“Anticipated healthcare stigma” (ever vs never)		“Enacted general social stigma” (ever vs never)	
*n* = *5544 unless otherwise specified*	Unadjusted OR (95% CI)	Adjusted OR (95% CI)	Unadjusted OR (95% CI)	Adjusted OR (95% CI)	Unadjusted OR (95% CI)	Adjusted OR (95% CI)
Median age (per 10 years)	0.98 (0.93, 1.05)	0.94 (0.87, 1.02)	1.06 (0.97, 1.15)	1.11 (1.00, 1.24)	0.97 (0.91, 1.03)	0.97 (0.90, 1.05)
Education						
Secondary	1.00	1.00	1.00	1.00	1.00	1.00
Incomplete higher	1.06 (0.91, 1.24)	1.06 (0.91, 1.25)	0.93 (0.75, 1.15)	0.92 (0.75, 1.15)	0.89 (0.76, 1.03)	0.86 (0.73, 1.00)
Complete higher	1.07 (0.93, 1.22)	1.06 (0.91, 1.23)	0.82 (0.67, 1.00)	0.79 (0.64, 0.98)	0.89 (0.77, 1.01)	0.84 (0.73, 0.98)
Official marital status						
Never married	1.00	1.00	1.00	1.00	1.00	1.00
Officially married	0.35 (0.25, 0.49)	0.72 (0.41, 1.29)	0.82 (0.56, 1.21)	0.72 (0.36, 1.47)	0.49 (0.37, 0.67)	1.30 (0.75, 2.25)
Divorced or widowed	1.06 (0.89, 1.26)	1.12 (0.90, 1.38)	1.02 (0.80, 1.29)	0.74 (0.55, 0.98)	0.90 (0.76, 1.08)	0.95 (0.77, 1.18)
Cohabitation status						
Live with parents/relatives	0.91 (0.79, 1.04)	0.94 (0.81, 1.08)	0.90 (0.75, 1.08)	0.90 (0.73, 1.10)	0.87 (0.76, 0.99)	0.84 (0.73, 0.97)
Live alone	1.00	1.00	1.00	1.00	1.00	1.00
Live with male partner	1.41 (1.19, 1.66)	1.29 (1.09, 1.53)	0.99 (0.78, 1.26)	1.11 (0.87, 1.42)	1.12 (0.94, 1.32)	1.00 (0.85, 1.19)
Live with female partner	0.33 (0.23, 0.47)	0.53 (0.29, 0.95)	0.80 (0.54, 1.18)	0.72 (0.36, 1.47)	0.40 (0.29, 0.55)	0.41 (0.23, 0.72)
Ever imprisoned						
Don’t report ever being imprisoned	1.00	1.00	1.00	1.00	1.00	1.00
Previously imprisoned	1.88 (1.36, 2.59)	2.15 (1.53, 3.02)	2.19 (1.49, 3.23)	2.05 (1.38, 3.06)	1.87 (1.35, 2.59)	2.12 (1.51, 2.98)
Sexual orientation						
Homosexual	1.00	1.00	1.00	1.00	1.00	1.00
Bisexual or other	0.76 (0.66, 0.86)	0.92 (0.80, 1.06)	1.33 (1.12, 1.58)	1.39 (1.15, 1.68)	0.72 (0.64, 0.82)	0.85 (0.74, 0.98)
Sexual orientation concealment						
Did not report any concealment	1.00	1.00	1.00	1.00	1.00	1.00
Partial concealment	0.59 (0.48, 0.72)	0.63 (0.51, 0.77)	1.14 (0.82, 1.57)	1.16 (0.84, 1.62)	0.69 (0.57, 0.85)	0.74 (0.60, 0.91)
Conceal from everyone	0.33 (0.27, 0.42)	0.39 (0.31, 0.49)	1.44 (1.02, 2.02)	1.45 (1.02, 2.06)	0.38 (0.30, 0.48)	0.43 (0.34, 0.55)
Client of MSM-focused NGO						
Does not report as being an NGO client	1.00	1.00	1.00	1.00	1.00	1.00
NGO client	1.21 (1.05, 1.39)	1.09 (0.94, 1.26)	0.91 (0.74, 1.11)	0.95 (0.77, 1.18)	1.31 (1.14, 1.51)	1.21 (1.04, 1.39)

*MSM* men who have sex with men; *OR* odds ratio; *95% CI* 95% confidence interval; *NGO* non-governmental organisation

^a^From mixed-effects logistic regression, with city as the random-effect

^b^The question available in the questionnaire was “Do you suppress the fact that you have sex with men?”. The possible answers were “Suppress this from everybody”, “Do not suppress this and is ready to say it anywhere”, and “Do note suppress this, but I will not talk about this first”

### Associations of Stigma with Demographics, Sexual Behaviors and HIV Disclosures

Table 3 reports unadjusted and adjusted odds ratios of the measures of stigma with dependent variables regarding: sexual orientation concealment, NGO client status, and sexual behaviors, HIV testing, HIV infection, and HIV treatment. MSM reporting stigma from family and friends and general social stigma had lower odds of concealing their sexual orientation from everyone, whilst those reporting anticipated healthcare stigma had higher odds. MSM that reported general social stigma were more likely to be members of an NGO. MSM reporting stigma from family and friends or general social stigma had lower ages of first sexual encounters with men. MSM reporting each stigma measure had higher odds of having had anal sex with a man in the last 6 months, had had more male anal sex partners in the last 30 days, and had lower odds of always using a condom for anal sex and of using a condom during their previous anal sexual intercourse. For each stigma measure, MSM reporting stigma had higher odds of having ever been renumerated for sex, having had group sex in the last 6 months, and having had chemsex in the last 30 days. MSM reporting general social stigma had lower odds of considering it to be very important to know of their sexual partners’ HIV status, whilst MSM reporting stigma from family and friends or general social stigma were less likely to consider disclosing their HIV status to be very important. For each stigma measure, MSM reporting stigma had lower odds of being aware of their last permanent sexual partner’s HIV status.

**Table 3:** Unadjusted and adjusted odds ratios (ORs) for HIV-related risk behaviors, testing, serostatus, or treatment by ever (versus never) having experienced each indicated stigma measure

Dependent variable	Stigma measures (independent variable)					
	“Stigma from family and friends” (ever vs never)		“Anticipated healthcare stigma” (ever vs never)		“Enacted general social stigma” (ever vs never)	
*n* = *5544 unless otherwise specified*	Unadjusted OR^a^ (95% CI)	Adjusted OR^a^ (95% CI)	Unadjusted OR^a^ (95% CI)	Adjusted OR^a^ (95% CI)	Unadjusted OR^a^ (95% CI)	Adjusted OR^a^ (95% CI)
Sexual orientation concealed from everyone	0.52 (0.45, 0.60)	0.58 (0.50, 0.67)	1.28 (1.07, 1.54)	1.26 (1.04, 1.52)	0.52 (0.45, 0.60)	0.56 (0.49, 0.65)
Client of NGO which provides prevention services to MSM	1.20 (1.04, 1.38)	1.09 (0.94, 1.26)	0.91 (0.74, 1.12)	0.96 (0.78, 1.19)	1.30 (1.13, 1.50)	1.22 (1.06, 1.41)
Age at first oral or anal sex with man (years) (*n* = *5470*)^b^	0.56 (0.44, 0.73)	0.72 (0.57, 0.89)	1.36 (0.95, 1.93)	1.17 (0.86, 1.58)	0.44 (0.34, 0.57)	0.61 (0.49, 0.76)
Had anal sex with a man in the last 6 months	1.28 (1.03, 1.59)	1.19 (0.96, 1.49)	1.30 (0.94, 1.79)	1.39 (1.00, 1.92)	1.82 (1.45, 2.28)	1.76 (1.40, 2.21)
Number of anal intercourses in last 30 days (*n* = *5400*)^c^	1.17 (1.10, 1.25)	1.09 (1.03, 1.16)	1.14 (1.04, 1.24)	1.17 (1.08, 1.28)	1.20 (1.13, 1.27)	1.14 (1.07, 1.21)
Number of male anal sex partners in last 30 days (*n* = *5519*)^c^	1.18 (1.11, 1.25)	1.14 (1.07, 1.21)	1.24 (1.14, 1.35)	1.24 (1.14, 1.34)	1.36 (1.28, 1.44)	1.32 (1.24, 1.40)
Report always using a condom for male anal sex during last 30 days (*of n* = *4733 who had anal sex with a man in last 30 days*)	0.65 (0.57, 0.74)	0.72 (0.63, 0.82)	0.72 (0.61, 0.87)	0.72 (0.59, 0.86)	0.50 (0.44, 0.57)	0.52 (0.45, 0.59)
Report using a condom for last anal sex with a man (*of n* = *5023 who had anal sex with a man in last 6 months*)	0.73 (0.63, 0.84)	0.81 (0.70, 0.94)	0.82 (0.67, 0.99)	0.80 (0.65, 0.98)	0.57 (0.49, 0.65)	0.59 (0.51, 0.68)
Ever renumerated for sex (*of n* = *5023 who had male anal in last 6 months*)	2.01 (1.71, 2.36)	1.92 (1.63, 2.27)	1.82 (1.47, 2.24)	1.85 (1.49, 2.30)	2.62 (2.23, 3.08)	2.50 (2.11, 2.95)
Report having group sex in the last 6 months	1.29 (1.11, 1.51)	1.20 (1.03, 1.41)	1.32 (1.07, 1.62)	1.32 (1.07, 1.63)	1.56 (1.35, 1.82)	1.47 (1.26, 1.72)
Report having chemsex in last 30 days	1.45 (1.13, 1.86)	1.36 (1.05, 1.76)	1.45 (1.05, 2.02)	1.39 (1.00, 1.94)	1.77 (1.38, 2.26)	1.66 (1.29, 2.14)
Considers knowing sexual partners’ HIV status to be very important (*n* = *5512*)	0.95 (0.84, 1.07)	0.93 (0.82, 1.05)	1.15 (0.97, 1.36)	1.17 (0.98, 1.38)	0.74 (0.66, 0.83)	0.73 (0.64, 0.82)
Considers disclosing HIV status to sexual partners be very important (*n* = *5492*)	0.87 (0.76, 0.98)	0.82 (0.73, 0.94)	1.00 (0.84, 1.19)	1.01 (0.85, 1.20)	0.71 (0.62, 0.80)	0.67 (0.59, 0.76)
Aware of last permanent partner’s HIV status (*of n* = *5027 who report having had a permanent sexual partner*)	0.99 (0.87, 1.12)	0.86 (0.75, 0.98)	0.80 (0.67, 0.95)	0.84 (0.70, 1.01)	0.85 (0.75, 0.96)	0.75 (0.65, 0.85)
Ever HIV tested	1.22 (1.07, 1.38)	1.05 (0.91, 1.21)	0.91 (0.77, 1.09)	0.95 (0.78, 1.16)	1.20 (1.06, 1.36)	1.06 (0.92, 1.22)
HIV tested in last year	1.11 (0.98, 1.26)	1.01 (0.88, 1.15)	0.96 (0.81, 1.14)	1.03 (0.85, 1.24)	1.13 (1.00, 1.28)	1.01 (0.88, 1.15)
Self-reporting HIV positive (*of n* = *3132 ever tested who disclosed HIV status*)	0.94 (0.64, 1.38)	0.92 (0.62, 1.36)	1.95 (1.20, 3.15)	1.91 (1.16, 3.14)	1.36 (0.95, 1.96)	1.29 (0.88, 1.87)
Testing HIV positive	0.88 (0.67, 1.16)	0.85 (0.64, 1.12)	1.29 (0.91, 1.83)	1.31 (0.92, 1.87)	1.22 (0.94, 1.58)	1.12 (0.86, 1.47)
Registered at AIDS centre (*of n* = *286 testing HIV positive*)	1.15 (0.63, 2.09)	1.02 (0.54, 1.90)	1.70 (0.81, 3.60)	1.35 (0.61, 2.95)	1.11 (0.64, 1.93)	1.05 (0.59, 1.85)
Receiving ART (*of n* = *286 testing HIV positive*)	1.14 (0.62, 2.11)	1.06 (0.56, 2.00)	1.52 (0.70, 3.30)	1.27 (0.56, 2.86)	1.03 (0.58, 1.81)	0.99 (0.55, 1.78)

*HIV* human immunodeficiency virus; *OR* odds ratio; *95% CI* 95% confidence interval; *AIDS* acquired immune deficiency syndrome; *ART* antiretroviral treatment

^a^Unless otherwise specified, odds ratios (with 95% confidence intervals) from mixed-effects logistic regression, with city as the random-effect, are displayed. Adjusted analyses contained variables on age, education level, ever-imprisoned, marital status, cohabitation status, sexual-orientation, sexual-orientation concealment, and being an NGO client

^b^Beta coefficient (with 95% confidence intervals) from mixed-effects linear regression, with city as the random-effect

^c^Incidence rate ratio (with 95% confidence intervals) and p value from mixed-effects negative binomial regression, with city as the random-effect

### Associations of Stigma with HIV Testing, Status, and Treatment

Table 3 also reports unadjusted and adjusted odds ratios of stigma with HIV testing, HIV infection, and HIV treatment. There were few associations between any of the stigma measures and these variables, including having been tested for HIV ever or in the prior year (among MSM without HIV). MSM with anticipated healthcare stigma had higher odds of self-reporting their awareness of having HIV, but this association was not seen with the HIV test results. Among those with HIV, there were no associations between stigma and being registered at an AIDS centre or receiving ART.

## Discussion

Our finding that a sizeable proportion of MSM in Ukraine have experienced some form of sexual behavior stigma across their lifetimes is consistent with findings in other settings [15, 26]. Particularly, vulnerable populations, including those less educated or ever imprisoned, experienced more stigma than those with higher levels of education or who had never been imprisoned. Our analysis of data from a national IBBS demonstrates that ever experiencing sexual behavior stigma is associated with greater levels of sexual risk behaviors among Ukrainian MSM. Associations were relatively consistent across multiple forms of sexual behavior stigma and for multiple sexual risk behaviors. For example, the three included sexual behavior stigma measures were all independently associated with having more anal sex partners last month, as well as with being less likely to have used condoms at last anal intercourse. However, sexual behavior stigma was not associated with HIV infection, HIV testing, or HIV treatment in our analyses.

### HIV Risk-Behaviors and HIV Infection

In general, the three measures of lifetime stigma behaved similarly, and all three measures were associated with multiple sexual risk behaviors. Generally, fewer associations were observed between perceived or anticipated stigma and sexual risk behaviors than for the enacted stigma measure. Much of the literature focuses on internalised stigma [8], so this iterates the value of considering alternative types of stigma.

Our finding that sexual behavior stigma associates with HIV risk behaviors among MSM has been found elsewhere [14]. In other settings, sexual behavior stigma is associated with increased rates of UAI [26–28], concurrent sex partners [26–28], and being reimbursed for sex [26, 29]. Our finding that sexual behavior stigma is negatively associated with discussing/valuing discussing HIV status with sex partners contrasts findings from a Europe-wide internet survey [13]. This could be because we measured sexual behavior stigma at the individual-level rather than country-level stigma which includes legislation and general population attitudes towards sexual minorities [13]. Our study builds on the literature by demonstrating associations between HIV risk behaviors with community-level sexual behavior stigma in an understudied Eastern European context.

Paradoxically, ever receiving sexual-behavior stigma was not associated with testing HIV seropositive in our analysis despite associations with numerous HIV-related sexual risk-behaviors. Notably, HIV seropositivity was the only variable not measured by self-report in this study and therefore the only variable not subject to recall bias. The apparent discrepancy between stigma associating with HIV risk-behavior but not HIV seropositivity may reduce confidence in the risk-behavior results. A potential explanation is that behaviors may change following a HIV diagnosis, or, alternatively, this could be due to a lack of statistical power due to there being 286 MSM testing positive for HIV. We cannot rule out that associations between stigma and risk-behaviors arose from unmeasured confounding or biases, such as recall or reporting bias. However, in cross-sectional analyses of IBBSs in other settings sexual-behavior stigma did associate with prevalent HIV [30, 31]. Furthermore, ever experiencing sexual-behavior stigma has been found to longitudinally associate with incidence of a combined measure of sexually transmitted infections and HIV in Nigeria, although it could not be assessed whether this association held for HIV incidence independently [28]. These studies utilised similar items to the Ukrainian IBBS but created different measures of stigma and were in sub-Saharan African settings, which might explain differential results [28, 30, 31].

### HIV Testing and Treatment

Associations between stigmas experienced by MSM with HIV testing and treatment are noted by other studies [6, 14]. However, there is significant heterogeneity in constructs measured, varying by stigmatised characteristic (e.g. sexual behavior, sexual orientation, HIV stigma), form (e.g. internalised, enacted) and level (e.g. community-level, structural-level) [7]. Therefore, lack of associations between HIV-testing or treatment with specifically community-level sexual behavior stigma in Ukraine may not necessarily indicate divergence from the literature.

Multi-country studies have found associations between intersecting stigmas relating to particular sexual practices as well as stigma relating to sexual-orientation with accessibility of HIV-testing services for MSM [32, 33]. However, several single-country studies have failed to observe associations between community-level sexual behavior stigma and actual HIV testing behaviors, supported by our results in Ukraine [19, 34]. Elsewhere internalised sexual orientation stigma has been found to associate with HIV-testing in multivariate analyses [35, 36]. Therefore, internalised sexual orientation stigma, unmeasured here, might be the important stigma construct for HIV testing behavior [35]. Interestingly, our three measures of enacted stigma associated with increased HIV testing behaviors in univariable analyses but attenuated on adjustment. This could reflect known associations between being an NGO client and HIV testing in Ukraine [22]. In our analyses being an NGO client generally associated with receipt of enacted stigma. This could be because NGOs are successfully reaching at-risk populations, or because those that are more willing to disclose their MSM status are accessing NGOs. Alternatively, MSM may be ‘outed’ by being an NGO client which could lead to experiences of stigma or that clients of NGOs are more aware of stigma. NGO contact might therefore result in univariate associations between enacted stigmas and increased HIV testing which attenuate following adjustment on NGO client status.

Our analyses did not identify associations between sexual behavior stigma and HIV treatment among HIV seropositive MSM. However, the small crude number of seropositive MSM in our sample mean our analyses may be underpowered to find such associations. In multi-country studies reported accessibility of HIV treatment negatively associates with sexual behavior stigma among MSM, and HIV stigma among HIV positive MSM [33], but this does not constitute direct evidence of reduced utilisation.

### Strengths and Limitations

A key strength of our analyses is a large sample size of > 5500 MSM with survey data from 26 Ukrainian cities spanning all regions of Ukraine. However, the median age of included MSM was 27 indicating a significantly younger age profile than the general population of men in Ukraine [22]. This is a concern because older men may have experienced more stigma and consequently be more hidden, biasing estimations of stigma, or potentially may have experienced no stigma because they are more hidden. RDS sampling drew upon initial seeds in each city, potentially biasing the sample towards participants with characteristics similar to the initial seeds. We did not use RDS weights to directly adjust for the sampling method although we did account for clustering at the city level. Excluding observations with missing stigma data also risks biasing results, although the low proportion of excluded results means any effect is likely limited.

Importantly, the cross-sectional study design means the temporal ordering of variables in the dataset cannot be confirmed. Causality is therefore difficult to assess, and we can only consider associations, for example it is possible sexual behavior stigma followed sexual risk behaviors or other variables of interest rather than preceding them. Several self-reported variables were asked as lifetime experiences, including stigma, further obfuscating temporal ordering. While HIV seropositivity results were measured biologically, all other variables were self-reported so potentially subject to varying levels of bias. Participant responses, including refusal to answer questions, may be subject to social desirability bias which could plausibly differentially affect those who had and had not experienced stigma. Participants were asked about past behaviors and experiences over timeframes ranging from months to ever, so recall bias could influence results. Inconsistent timeliness of variables could also introduce recency bias. ‘Ever’ variables such as stigma could greatly precede included dependent variables for any given observation, and so may be less likely to be recollected. Future studies should look to collect more detailed measures of stigma, included over more recent time-periods.

It important to note that this study did not utilise a measure of sexual behavior stigma validated for Ukrainian MSM. It is not clear whether the survey items reflect the intended concepts in Ukraine, though they have been used in several settings in the United States and sub-Saharan Africa [8, 15]. However, the development of scales/measures that are contextually appropriate but also allow comparisons globally remains a challenge for the field. Notably, significant heterogeneity among stigma constructs used in other studies of sexual behavior stigma makes it difficult for us to meaningfully compare our findings [7]. Also, lack of observed associations with our stigma measures cannot preclude associations with forms of stigma not surveyed in the IBBS, as discussed for internalised stigma and HIV testing [28].

Finally, the data used in this analysis are from 2017 before the launch of the full invasion by Russia in 2022 and the impacts of this are unknown. The start of the war in 2014, including the annexation of Crimea, saw large increases in homophobic violence and hate crimes in Crimea and Eastern Ukraine, causing many MSM to flee to Western Ukraine [37]. The first few months of the full invasion has seen reports of homophobic and transphobic violence [38], in some cases perpetuated by the territorial defense. There are fears that Russia will impose oppressive laws in the occupied regions, which significantly impacted the mental health of MSM when passed in Russia [39]. However, there is also hope that the conflict, which has united the country against Russian aggression, may lead to increased advocacy capacity for the rights of Lesbian, Gay, Bisexual, Transgender, Queer, and Intersex (LGBTQI) people living in Ukraine [38].

## Conclusions

Ukraine is experiencing a sizeable and sustained HIV epidemic and HIV prevalence remains high among Ukrainian MSM [2, 20]. Our findings demonstrate that associations between sexual behavior stigma and increased HIV risk behaviors apply in an understudied Eastern European context. However, we did not observe associations between sexual behavior stigma with HIV-testing, treatment, and, notably, with HIV infection. Future research is warranted to elucidate the relationship between stigma, risk behavior and HIV infection in Ukraine. Particularly, there is need for longitudinal study designs to enable assessment of causality of stigma and its potential adverse effects on HIV-outcomes [6]. There is also a need to measure changes in stigma over time, including over the course of the ongoing conflict with Russia.

### Supplementary Information

Below is the link to the electronic supplementary material.

Supplementary file1 (DOCX 88 KB)
