# Supplementary material for: Associations Between Sexual Behavior Stigma and HIV Risk Behaviors, Testing, Treatment, and Infection Among Men Who have Sex with Men in Ukraine
Source: AIDS Behav. 2023 Oct 4;28(3):786–98. doi: 10.1007/s10461-023-04182-1 (PMC10896872; doi:10.1007/s10461-023-04182-1)
Supplement: Supplementary file 1 — Supplementary file1 (DOCX 88 KB) [file 10461_2023_4182_MOESM1_ESM.docx]

**Associations between sexual behavior stigma and HIV risk behaviors, testing, treatment, and infection among men who have sex with men in Ukraine**

**Supplementary materials**

**Supplementary table 1:** Participating cities

| **#** | **City** | **Implemented sample** |
| --- | --- | --- |
| 1 | Vinnytsia | 150 |
| 2 | Dnipro | 250 |
| 3 | Donetsk | 250 |
| 4 | Mariupol | 250 |
| 5 | Zhytomyr | 200 |
| 6 | Zaporizhzhya | 250 |
| 7 | Ivano-Frankivsk | 218 |
| 8 | Kyiv | 249 |
| 9 | Bila Tserkva | 200 |
| 10 | Kropyvnytskyi | 150 |
| 11 | Lutsk | 200 |
| 12 | Lviv | 250 |
| 13 | Mykolayiv | 150 |
| 14 | Odesa | 250 |
| 15 | Poltava | 201 |
| 16 | Rivne | 150 |
| 17 | Sevastopol | 300 |
| 18 | Simferopol | 251 |
| 19 | Sumy | 150 |
| 20 | Ternopil | 150 |
| 21 | Uzhhorod | 250 |
| 22 | Kharkiv | 352 |
| 23 | Kherson | 150 |
| 24 | Khmelnytskyi | 150 |
| 25 | Cherkasy | 250 |
| 26 | Chernivtsi | 300 |
| 27 | Chernihiv | 300 |
| Total | | 5.971 |

**Generating stigma categories**

**Supplementary table 2:** Variables related to stigma

| **Stigma variable** | **#** | **"No"**  **(%)** | **"Yes" (%)** | **“Don’t know (%)** |
| --- | --- | --- | --- | --- |
| Have you ever felt excluded from family activities because you have sex with men? | **1** | 90.86 | 8.83 | 0.31 |
| Have you ever felt that family members have made discriminatory remarks or gossiped about you because you have sex with men? | **2** | 81.78 | 17.69 | 0.53 |
| Have you ever felt rejected by your friends because you have sex with men? | **3** | 79.13 | 20.44 | 0.43 |
| Have you ever felt afraid to go to healthcare services because you worry someone may learn you have sex with men? | **4** | 87.68 | 11.99 | 0.33 |
| Have you ever avoided going to health care services because you worry someone may learn you have sex with men? | **5** | 92.24 | 7.47 | 0.29 |
| Have you ever felt that you were not treated well in a health center because someone knew that you have sex with men? | **6** | 95.94 | 3.61 | 0.45 |
| Have you ever heard health care providers gossing about you because you have sex with men? | **7** | 94.94 | 4.56 | 0.5 |
| Have you ever felt that the police refused to protect you because you have sex with men? | **8** | 96.61 | 2.58 | 0.81 |
| Have you ever felt scared to be in public places because you have sex with men? | **9** | 88.73 | 10.51 | 0.76 |
| Have you ever been verballed harassed and felt it was because you have sex with men? | **10** | 72.9 | 26.88 | 0.22 |
| Have you ever been blackmailed by someone because you have sex with men? | **11** | 93.32 | 6.52 | 0.15 |
| Has someone ever physically hurt you because you have sex with men? | **12** | 91.14 | 8.57 | 0.29 |
| Have you ever been forced to have sex when you did not want to relating to the fact that you have sex with men? | **13** | 93.91 | 5.39 | 0.71 |

**Supplementary table 3:** Creating stigma categories

|  |  | **"Never"** |  | **"Ever"** |  |
| --- | --- | --- | --- | --- | --- |
| **Stigma composite variable** | **Items** | n | column % | n | column % |
| "Stigma from family and friends" | **1,2,3** | 3,881 | 70 | 1,663 | 30 |
| "Anticipated healthcare stigma" | **4,5** | 4,846 | 87.41 | 698 | 12.59 |
| "General social stigma"* | **6,7,8,10,11,12,13** | 3,739 | 67.44 | 1,805 | 32.56 |

*(enacted version with item 9 removed)

**Supplementary table 4:** Characteristics of MSM by whether they have experienced each stigma measure

|  | **"Stigma from family and friends"** | | | | **"Anticipated healthcare stigma"** | | | | **"Enacted general social stigma"** | | | |
| --- | --- | --- | --- | --- | --- | --- | --- | --- | --- | --- | --- | --- |
|  | Never | | Ever | | Never | | Ever | | Never | | Ever | |
| **Variable** | (n= 3881, 70.0 %) | | (n= 1663, 30.0%) | | (n= 4846, 87.4%) | | (n= 698, 12.6%) | | (n= 3739, 67.4%) | | (n= 1805, 32.6%) | |
| *n= 5544 unless otherwise specified* | n | column % | n | column % | n | column % | n | column % | n | column % | n | column % |
|  |  |  |  |  |  |  |  |  |  |  |  |  |
| **Median age** (interquartile range) *^a^* | 27 (21–35) | | 27 (21–35) | | 27 (21–35) | | 27 (21–36) | | 27 (21–35) | | 27 (21–34) | |
| **Education** |  |  |  |  |  |  |  |  |  |  |  |  |
| Secondary | 1670 | 43.0% | 709 | 42.6% | 2047 | 42.2% | 332 | 47.6% | 1544 | 41.3% | 835 | 46.3% |
| Incomplete higher | 946 | 24.4% | 408 | 24.5% | 1188 | 24.5% | 166 | 23.8% | 954 | 25.5% | 400 | 22.2% |
| Complete higher | 1265 | 32.6% | 546 | 32.8% | 1611 | 33.2% | 200 | 28.7% | 1241 | 33.2% | 570 | 31.6% |
| **Official Marital Status** |  |  |  |  |  |  |  |  |  |  |  |  |
| Never married | 3122 | 80.4% | 1383 | 83.2% | 3945 | 81.4% | 560 | 80.2% | 2996 | 80.1% | 1509 | 83.6% |
| Officially married | 252 | 6.5% | 41 | 2.5% | 259 | 5.3% | 34 | 4.9% | 231 | 6.2% | 62 | 3.4% |
| Divorced or widowed | 507 | 13.1% | 239 | 14.4% | 642 | 13.3% | 104 | 14.9% | 512 | 13.7% | 234 | 13.0% |
| **Cohabitation status** |  |  |  |  |  |  |  |  |  |  |  |  |
| Live with parents/relatives | 1524 | 39.3% | 608 | 36.6% | 1870 | 38.6% | 262 | 37.5% | 1467 | 39.2% | 665 | 36.8% |
| Live alone | 1552 | 40.0% | 668 | 40.2% | 1933 | 39.9% | 287 | 41.1% | 1474 | 39.4% | 746 | 41.3% |
| Live with male partner | 557 | 14.4% | 349 | 21.0% | 791 | 16.3% | 115 | 16.5% | 564 | 15.1% | 342 | 19.0% |
| Live with female partner | 248 | 6.4% | 38 | 2.3% | 252 | 5.2% | 34 | 4.9% | 234 | 6.3% | 52 | 2.9% |
| **Ever imprisoned** |  |  |  |  |  |  |  |  |  |  |  |  |
| Don't report ever being imprisoned | 3789 | 97.6% | 1588 | 95.5% | 4719 | 97.4% | 658 | 94.3% | 3651 | 97.7% | 1726 | 95.6% |
| Previously imprisoned | 92 | 2.4% | 75 | 4.5% | 127 | 2.6% | 40 | 5.7% | 88 | 2.4% | 79 | 4.4% |
| **Sexual orientation** |  |  |  |  |  |  |  |  |  |  |  |  |
| Homosexual | 2443 | 63.0% | 1140 | 68.6% | 3182 | 65.7% | 401 | 57.5% | 2326 | 62.2% | 1257 | 69.6% |
| Bisexual or other | 1438 | 37.1% | 523 | 31.5% | 1664 | 34.3% | 297 | 42.6% | 1413 | 37.8% | 548 | 30.4% |
| **Sexual orientation concealment*** |  |  |  |  |  |  |  |  |  |  |  |  |
| Did not report any concealment | 262 | 6.8% | 212 | 12.8% | 426 | 8.8% | 48 | 6.9% | 257 | 6.9% | 217 | 12.0% |
| Do not conceal, but will not talk about this first | 2387 | 61.5% | 1095 | 65.8% | 3079 | 63.5% | 403 | 57.7% | 2271 | 60.7% | 1211 | 67.1% |
| Conceal from everyone | 1232 | 31.7% | 356 | 21.4% | 1341 | 27.7% | 247 | 35.4% | 1211 | 32.4% | 377 | 20.9% |
| **Client of NGO which provides prevention services to MSM** |  |  |  |  |  |  |  |  |  |  |  |  |
| Does not report as being an NGO client | 2871 | 74.0% | 1145 | 68.9% | 3493 | 72.1% | 523 | 74.9% | 2798 | 74.8% | 1218 | 67.5% |
| NGO client | 1010 | 26.0% | 518 | 31.2% | 1353 | 27.9% | 175 | 25.1% | 941 | 25.2% | 587 | 32.5% |
| **Median age at first oral or anal sex with man** *(n=5470) (interquartile range) ^a^* | 17 (16–20) | | 17 (15–19) | | 17 (15–19) | | 17 (15–20) | | 17 (16–20) | | 17 (15–19) | |
| **Methods to seek male sexual partners in last 6 months** |  |  |  |  |  |  |  |  |  |  |  |  |
| Don't report using internet/mobile apps/teletext to find partners | 806 | 20.8% | 352 | 21.2% | 964 | 19.9% | 194 | 27.8% | 784 | 21.0% | 374 | 20.7% |
| Used internet/mobile apps/teletext to find partners | 2599 | 67.0% | 1055 | 63.4% | 3229 | 66.6% | 425 | 60.9% | 2494 | 66.7% | 1160 | 64.3% |
| Did not seek male partners in last 6m | 476 | 12.3% | 256 | 15.4% | 653 | 13.5% | 79 | 11.3% | 461 | 12.3% | 271 | 15.0% |
| **Had anal sex with a man in the last 6 months** |  |  |  |  |  |  |  |  |  |  |  |  |
| Did not report having had anal sex with man last 6 months | 391 | 10.1% | 130 | 7.8% | 474 | 9.8% | 47 | 6.7% | 405 | 10.8% | 116 | 6.4% |
| Yes - Reported having had anal sex with man last 6 months | 3490 | 89.9% | 1533 | 92.2% | 4372 | 90.2% | 651 | 93.3% | 3334 | 89.2% | 1689 | 93.6% |
| **Median number anal intercourses in last 30 days** *(n=5400) (interquartile range)^a^* | 4 (2–8) | | 5 (2–10) | | 4 (2–8) | | 4 (2–9) | | 4 (2–8) | | 5 (2–10) | |
| **Median number male anal sex partners in last 30 days** *(n=5519) (interquartile range)^a^* | 1 (1–3) | | 1 (1–3) | | 1 (1–3) | | 1 (1–4) | | 1 (1–3) | | 1 (1–3) | |
| **Condom use for anal sex last 30 days** |  |  |  |  |  |  |  |  |  |  |  |  |
| Never | 295 | 7.6% | 188 | 11.3% | 406 | 8.4% | 77 | 11.0% | 269 | 7.2% | 214 | 11.9% |
| Sometimes | 940 | 24.2% | 526 | 31.6% | 1251 | 25.8% | 215 | 30.8% | 866 | 23.2% | 600 | 33.2% |
| Always | 2041 | 52.6% | 743 | 44.7% | 2471 | 51.0% | 313 | 44.8% | 2057 | 55.0% | 727 | 40.3% |
| Didn't have male anal sex last 30 days | 605 | 15.6% | 206 | 12.4% | 718 | 14.8% | 93 | 13.3% | 547 | 14.6% | 264 | 14.6% |
| **Condom use at last anal sex with man** |  |  |  |  |  |  |  |  |  |  |  |  |
| Didn't report having used condoms at last anal with man | 714 | 18.4% | 411 | 24.7% | 956 | 19.7% | 169 | 24.2% | 623 | 16.7% | 502 | 27.8% |
| Report using condoms at last anal with man | 2776 | 71.5% | 1122 | 67.5% | 3416 | 70.5% | 482 | 69.1% | 2711 | 72.5% | 1187 | 65.8% |
| Didn't report having had anal with man in last 6 months | 391 | 10.1% | 130 | 7.8% | 474 | 9.8% | 47 | 6.7% | 405 | 10.8% | 116 | 6.4% |
| **Ever renumerated for sex** |  |  |  |  |  |  |  |  |  |  |  |  |
| Didn't report ever having been renumerated for sex | 3053 | 78.7% | 1186 | 71.3% | 3741 | 77.2% | 498 | 71.4% | 2970 | 79.4% | 1269 | 70.3% |
| Yes - Reported ever having been renumerated for sex | 437 | 11.3% | 347 | 20.9% | 631 | 13.0% | 153 | 21.9% | 364 | 9.7% | 420 | 23.3% |
| Didn't report having had anal with man in last 6 months | 391 | 10.1% | 130 | 7.8% | 474 | 9.8% | 47 | 6.7% | 405 | 10.8% | 116 | 6.4% |
| **Group sex in last 6 months** |  |  |  |  |  |  |  |  |  |  |  |  |
| Don't report having had group sex | 3265 | 84.1% | 1341 | 80.6% | 4046 | 83.5% | 560 | 80.2% | 3190 | 85.3% | 1416 | 78.5% |
| Had group sex | 616 | 15.9% | 322 | 19.4% | 800 | 16.5% | 138 | 19.8% | 549 | 14.7% | 389 | 21.6% |
| **Chemsex in last 30 days** |  |  |  |  |  |  |  |  |  |  |  |  |
| Don't report having had chemsex | 3701 | 95.4% | 1554 | 93.5% | 4607 | 95.1% | 648 | 92.8% | 3581 | 95.8% | 1674 | 92.7% |
| Had chemsex | 180 | 4.6% | 109 | 6.6% | 239 | 4.9% | 50 | 7.2% | 158 | 4.2% | 131 | 7.3% |
| **Values knowing sexual partners HIV status** *(n=5512)* |  |  |  |  |  |  |  |  |  |  |  |  |
| Not important at all | 139 | 3.6% | 77 | 4.7% | 183 | 3.8% | 33 | 4.8% | 119 | 3.2% | 97 | 5.4% |
| Close to not important | 384 | 10.0% | 153 | 9.3% | 483 | 10.0% | 54 | 7.8% | 334 | 9.0% | 203 | 11.3% |
| Close to important | 1299 | 33.7% | 566 | 34.2% | 1656 | 34.4% | 209 | 30.1% | 1228 | 33.0% | 637 | 35.6% |
| Very important | 2037 | 52.8% | 857 | 51.9% | 2496 | 51.8% | 398 | 57.4% | 2039 | 54.8% | 855 | 47.7% |
| **Values disclosing HIV status to sexual partners** *(n=5492)* |  |  |  |  |  |  |  |  |  |  |  |  |
| Not important at all | 384 | 10.0% | 197 | 12.0% | 485 | 10.1% | 96 | 13.9% | 333 | 9.0% | 248 | 13.9% |
| Close to not important | 580 | 15.1% | 275 | 16.7% | 744 | 15.5% | 111 | 16.1% | 530 | 14.3% | 325 | 18.2% |
| Close to important | 1303 | 33.9% | 551 | 33.4% | 1658 | 34.5% | 196 | 28.4% | 1255 | 33.9% | 599 | 33.6% |
| Very important | 1577 | 41.0% | 625 | 37.9% | 1915 | 39.9% | 287 | 41.6% | 1589 | 42.9% | 613 | 34.3% |
| **Aware of last permanent partner's HIV status** |  |  |  |  |  |  |  |  |  |  |  |  |
| Don’t know his/her HIV status | 1462 | 37.7% | 649 | 39.0% | 1821 | 37.6% | 290 | 41.6% | 1375 | 36.8% | 736 | 40.8% |
| Know his/her HIV status | 2040 | 52.6% | 876 | 52.7% | 2574 | 53.1% | 342 | 49.0% | 2021 | 54.1% | 895 | 49.6% |
| Doesn't report having had a permanent sexual partner | 379 | 9.8% | 138 | 8.3% | 451 | 9.3% | 66 | 9.5% | 343 | 9.2% | 174 | 9.6% |
|  |  |  |  |  |  |  |  |  |  |  |  |  |
| **Ever HIV tested** |  |  |  |  |  |  |  |  |  |  |  |  |
| Don't report ever being HIV tested | 1394 | 35.9% | 525 | 31.6% | 1668 | 34.4% | 251 | 36.0% | 1355 | 36.2% | 564 | 31.3% |
| Ever HIV tested | 2487 | 64.1% | 1138 | 68.4% | 3178 | 65.6% | 447 | 64.0% | 2384 | 63.8% | 1241 | 68.8% |
| **HIV tested in last year** |  |  |  |  |  |  |  |  |  |  |  |  |
| Don't report being HIV-tested in last 12 months | 2214 | 57.1% | 913 | 54.9% | 2734 | 56.4% | 393 | 56.3% | 2149 | 57.5% | 978 | 54.2% |
| HIV tested in last 12 months | 1667 | 43.0% | 750 | 45.1% | 2112 | 43.6% | 305 | 43.7% | 1590 | 42.5% | 827 | 45.8% |
| **Self-reported HIV status** *(of n=3625 ever HIV-tested)* |  |  |  |  |  |  |  |  |  |  |  |  |
| HIV negative self-report | 2080 | 83.6% | 911 | 80.1% | 2662 | 83.8% | 329 | 73.6% | 1998 | 83.8% | 993 | 80.0% |
| HIV positive self-report | 101 | 4.1% | 40 | 3.5% | 117 | 3.7% | 24 | 5.4% | 85 | 3.6% | 56 | 4.5% |
| Declined to disclose HIV status | 306 | 12.3% | 187 | 16.4% | 399 | 12.6% | 94 | 21.0% | 301 | 12.6% | 192 | 15.5% |
| **HIV antibody (Ab) test result** |  |  |  |  |  |  |  |  |  |  |  |  |
| Negative | 3675 | 94.7% | 1583 | 95.2% | 4604 | 95.0% | 654 | 93.7% | 3562 | 95.3% | 1696 | 94.0% |
| Positive | 206 | 5.3% | 80 | 4.8% | 242 | 5.0% | 44 | 6.3% | 177 | 4.7% | 109 | 6.0% |
| **Registered at AIDS centre** *(of n=286 testing HIV positive)* |  |  |  |  |  |  |  |  |  |  |  |  |
| No | 117 | 56.8% | 44 | 55.0% | 137 | 56.6% | 24 | 54.6% | 100 | 56.5% | 61 | 56.0% |
| Yes | 89 | 43.2% | 36 | 45.0% | 105 | 43.4% | 20 | 45.5% | 77 | 43.5% | 48 | 44.0% |
| **Receiving ART** *(of n=286 testing HIV positive)* |  |  |  |  |  |  |  |  |  |  |  |  |
| No | 124 | 60.2% | 47 | 58.8% | 144 | 59.5% | 27 | 61.4% | 105 | 59.3% | 66 | 60.6% |
| Yes | 82 | 39.8% | 33 | 41.3% | 98 | 40.5% | 17 | 38.6% | 72 | 40.7% | 43 | 39.5% |

MSM, men who have sex with men; NGO, non-governmental organisation; HIV, human immunodeficiency virus; AIDS, acquired immune deficiency syndrome; ART, antiretroviral therapy.

1. Numerical variable therefore median and interquartile range are displayed instead of n and column percentage.

* The question available in the questionnaire was “Do you suppress the fact that you have sex with men?”. The possible answers were “Suppress this from everybody”, “Do not suppress this and is ready to say it anywhere”, and “Do note suppress this, but I will not talk about this first”.
